# Real-World Treatment Patterns and Clinical Effectiveness of Palbociclib Plus an Aromatase Inhibitor as First-Line Therapy in Advanced/Metastatic Breast Cancer: Analysis from the US Syapse Learning Health Network

**DOI:** 10.3390/curroncol29020089

**Published:** 2022-02-12

**Authors:** Jeanna Wallenta Law, Debanjali Mitra, Henry G. Kaplan, Tamuno Alfred, Adam M. Brufsky, Birol Emir, Haley McCracken, Xianchen Liu, Ronda G. Broome, Chenan Zhang, Caroline DiCristo, Connie Chen

**Affiliations:** 1 Syapse, San Francisco, CA 94107, USA; haley.huston@syapse.com (H.M.); ronda.broome@syapse.com (R.G.B.); chenan.zhang@syapse.com (C.Z.); 2 Pfizer Inc., New York, NY 10017, USA; debanjali.mitra@pfizer.com (D.M.); tamuno.alfred@pfizer.com (T.A.); birol.emir@pfizer.com (B.E.); jasonxc.liu@pfizer.com (X.L.); caroline.dicristo@pfizer.com (C.D.); connie.chen@pfizer.com (C.C.); 3 Swedish Cancer Institute, Seattle, WA 98104, USA; hank.kaplan@swedish.org; 4 Comprehensive Breast Cancer Center, University of Pittsburgh Medical Center, Pittsburgh, PA 15213, USA; brufskyam@upmc.edu

**Keywords:** HR+/HER2−, metastatic breast cancer, palbociclib, aromatase inhibitor, real-world data

## Abstract

This retrospective single-arm study assessed real-world treatment patterns and clinical outcomes in patients with hormone receptor—positive/human epidermal growth factor receptor 2—negative (HR+/HER2−) advanced/metastatic breast cancer (A/MBC) who received palbociclib plus an aromatase inhibitor as first-line therapy in US community health systems. Using electronic health records from the Syapse Learning Health Network, 242 patients were identified as having received first-line palbociclib plus an aromatase inhibitor between 3 February 2015, and 31 July 2019 (data cutoff 1 February 2020) resulting in a minimum potential 6-month follow-up period. In total, 56.6% of patients had de novo A/MBC at initial breast cancer diagnosis, 50.8% had bone-only disease, and 32.2% had visceral disease. Median follow-up was 22.4 months. Disease progression (26.4%) and intolerance/toxicity (14.9%) were the main reasons for treatment discontinuation. The median (95% CI) real-world progression-free survival was 31.7 (27.9—not estimable (NE)) months and 2-year estimated overall survival (OS) rate was 78.0%. In total, 25.6% of patients died; however, OS data are limited by the small population size and insufficient follow-up time. These real-world effectiveness outcomes complement findings from other real-world studies and randomized controlled trials and support palbociclib plus an aromatase inhibitor as first-line therapy for HR+/HER2− A/MBC.

## 1. Introduction

In 2022, it is estimated that 287,850 new cases of female breast cancer will be diagnosed, and the age-adjusted mortality rate is 20.1 per 100,000 women per year in the United States [1,2]. Approximately 6% of breast cancer cases are classified as advanced/metastatic breast cancer (A/MBC) at diagnosis, with the cancer having spread to distant organs and/or lymph nodes [3]. Diagnosis of A/MBC is associated with a poor prognosis as the 5-year survival rate among women with A/MBC is 29.0% [2].

Hormone receptor—positive/human epidermal growth factor receptor 2—negative (HR+/HER2−) breast cancer is the most common molecular subtype, accounting for 73% of all breast cancers [3]. The National Comprehensive Cancer Network guidelines recommend a cyclin-dependent kinase 4/6 (CDK4/6) inhibitor plus endocrine therapy as first-line therapy for patients with HR+/HER2− A/MBC [4]. Palbociclib, a CDK4/6 inhibitor, was approved for the treatment of HR+/HER2− A/MBC in combination with an aromatase inhibitor in February 2015 and in combination with fulvestrant in February 2016 [5,6,7]. In the United States, palbociclib approval was based on findings from the PALOMA clinical trial program. The PALOMA-1 [8] phase 2 study and the PALOMA-2 [9] phase 3 study assessed the efficacy of palbociclib plus letrozole versus letrozole alone or letrozole plus placebo, respectively, as initial endocrine-based therapy for estrogen receptor—positive (ER+)/HER2− A/MBC. PALOMA-3 assessed outcomes of palbociclib plus fulvestrant versus placebo plus fulvestrant in patients with HR+/HER2− A/MBC who progressed during prior endocrine therapy [10]. All three PALOMA trials demonstrated a significant improvement in median progression-free survival (PFS) with palbociclib combination therapy versus control groups [8,9,10,11,12]. Although not statistically significant, overall survival (OS) results showed that median OS was longer with palbociclib plus letrozole versus letrozole alone in PALOMA-1 (37.5 vs. 34.5 months; hazard ratio, 0.897 [95% CI, 0.623–1.294]; *p* = 0.281) and with palbociclib plus fulvestrant versus placebo plus fulvestrant in PALOMA-3 (34.8 vs. 28.0 months; hazard ratio, 0.81 [95% CI, 0.65–0.99]; *p* = 0.0221 at a median follow-up of 73.3 months) [13,14,15]. Median OS results for PALOMA-2 are not yet mature.

Palbociclib, the first CDK4/6 inhibitor approved to treat HR+/HER2− A/MBC, has been available for 7 years in the United States, facilitating the accumulation of real-world patient data. Real-world studies provide an opportunity to include diverse populations, such as elderly patients and patients with varying disease burdens, comorbidities, and different baseline performance status scores who may not be eligible for enrollment in clinical trials. Thus, real-world evidence can facilitate a deeper understanding of the treatment patterns, tolerability, and effectiveness of drug regimens in routine clinical practice and across a wide range of patients. Moreover, real-world evidence complements data from randomized controlled trials and is increasingly being evaluated by regulatory agencies [16]. 

This single-arm, retrospective study evaluated patient characteristics, treatment patterns, and clinical outcomes in patients with HR+/HER2− A/MBC who received palbociclib plus an aromatase inhibitor as first-line therapy in a real-world setting. This study adds to the existing body of literature regarding real-world palbociclib use by harnessing an electronic health record (EHR) system supplemented with chart review that includes a diverse patient population to evaluate palbociclib treatment patterns and clinical outcomes in A/MBC. This real-world data network is unique in that it includes a cohort of patients who are from large integrated community health systems and captures both oncology and non-oncology care data, whereas previous palbociclib real-world studies describe data from academic or other independent community care practices [17,18,19,20]. Patients included in this network are unlikely to be described in other EHR systems unless a patient left this EHR network to be treated in another EHR system. Overall, the data presented herein add to the robustness of real-world evidence from other EHR data sources, providing an additional perspective into the real-world effectiveness of palbociclib plus an aromatase inhibitor as first-line therapy for HR+/HER2− A/MBC.

## 2. Materials and Methods

### 2.1. Study Design and Data Source

**Figure 1:**
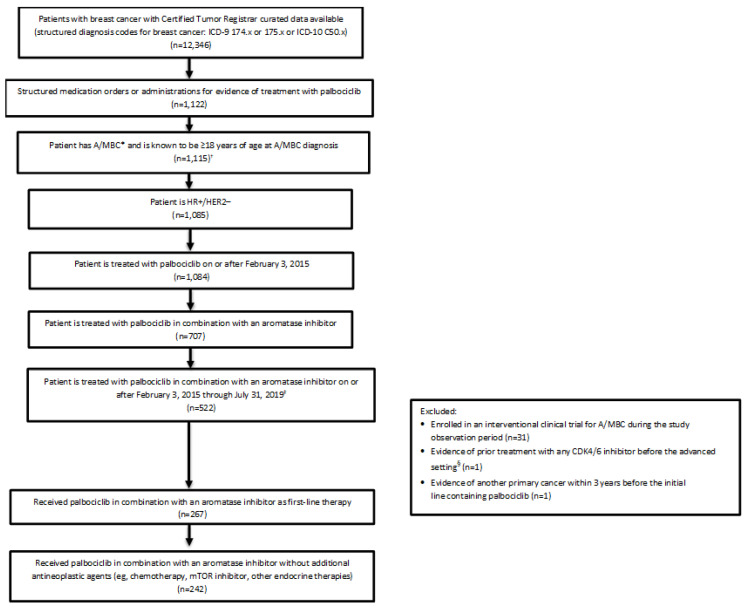
Patient attrition flowchart. A/MBC = advanced/metastatic breast cancer; CDK4/6 = cyclin-dependent kinase 4/6; HR+/HER2− = hormone receptor—positive/human epidermal growth factor receptor 2—negative; ICD-9/-10 = International Classification of Diseases, Ninth/Tenth Revision; mTOR = mechanistic target of rapamycin. * Advanced breast cancer defined as stage IIIb or IIIc or metastatic breast cancer at diagnosis. ^†^ The 7 patients excluded at this step were all confirmed to be ≥18 years old at initial breast cancer diagnosis, but not confirmed as having A/MBC. ^‡^ Study end date February 1, 2020. February 1, 2020 allows for the opportunity to have 6 months of follow up time after initiating palbociclib. ^§^ If a CDK4/6 inhibitor was administered (start date) 30 days prior to the A/MBC diagnosis date, the patient was excluded.

This single arm, retrospective study used the US Syapse Learning Health Network (LHN) to evaluate patient characteristics, treatment patterns, and clinical outcomes among patients receiving first-line therapy with palbociclib plus an aromatase inhibitor for A/MBC. The Syapse LHN is a longitudinal database capturing cancer care and noncancer care data and has previously been used by the US Food and Drug Administration [21,22,23]. It includes patients from large community health systems based in the United States that include managed care delivery networks across 25 states, 457 hospitals, and more than 1300 oncologists. The Syapse LHN point-of-care software platform collects comprehensive real-world data on a daily basis by gathering inpatient and outpatient data from a variety of sources, such as electronic medical records, an electronic data warehouse (which brings together data from multiple cancer-specific and noncancer-specific data sources in the healthcare system), a lab information system, a picture archiving and communication system, computerized physician order entries, and hospital-based cancer registries. All records underwent a chart abstraction by certified tumor registrars that was followed by a quality control review with evaluation for consistency, completeness, and outlier values by the Syapse epidemiology and clinical analytics teams. These data were used to identify patients with a breast cancer diagnosis International Classification of Diseases (ICD) code (ICD-9 174.x or 175.x or ICD-10 C50.x) as well as medication orders for palbociclib (Figure 1). A validated mortality endpoint was used for survival analyses [24]. The endpoint was a composite of death data collected from the Surveillance, Epidemiology, and End Results Program national cancer registry, Social Security Death Index, online obituary data from a third-party vendor, manually extracted data from online obituaries and physician notes, and the health system’s EHRs, including hospital-based cancer registries for date of death. 

Patients included were men or women ≥18 years of age with HR+/HER2− A/MBC (stage IIIB, IIIC, IV, or progressed to A/MBC from an earlier diagnosis) who initiated palbociclib plus an aromatase inhibitor as first-line therapy for A/MBC from 3 February 2015, to 31 July 2019. Exclusion criteria included enrollment in a clinical trial for A/MBC during the study period; evidence of another primary cancer within 3 years before starting first-line therapy; and evidence of prior CDK4/6 inhibitor treatment in the adjuvant setting. All patients who met the inclusion and exclusion criteria and had a date of first-line palbociclib plus aromatase inhibitor combination therapy initiation (index date) between 3 February 2015 and 31 July 2019 were evaluated; data cutoff was 1 February 2020, resulting in a minimum potential 6-month follow-up period. Aromatase inhibitor partners included letrozole, anastrozole, or exemestane. Combination treatment had to be initiated within 60 days, allowing for potential delays in access to either medication.

### 2.2. Regimens and Line of Therapy

A first-line regimen was defined as the first antineoplastic drug(s) a patient receives after diagnosis of an A/MBC, including all drugs received within a 60-day window of the first antineoplastic agent received. The start date of a first-line regimen was defined as the start of the first medication in the combination. The end of the first-line regimen was defined as the discontinuation of all antineoplastic treatments in the regimen and a gap of ≥90 days for each treatment before evidence of next treatment or the addition of a non-interchangeable, new antineoplastic after the first 60 days of first-line regimen (if added before 60-day cutoff, a new line of therapy was not triggered). Aromatase inhibitors were the only interchangeable drugs, meaning that switching between aromatase inhibitors did not trigger a new line. The end date of the first-line regimen was the stop date of the last antineoplastic treatment in the regimen (i.e., if palbociclib was discontinued first and the aromatase inhibitor second, the end of aromatase inhibitor treatment was recorded as the line of therapy end date).

### 2.3. Outcomes

The primary objectives were to describe key demographic and clinical characteristics captured at the time of A/MBC diagnosis, treatment patterns (i.e., dosing patterns, time to treatment discontinuation (TTD), time to chemotherapy (TTC)), and clinical effectiveness (i.e., real-world PFS (rwPFS), OS). 

#### 2.3.1. Clinical Characteristics

Menopausal status among women was captured if it was stated in the clinician notes or was based on the National Comprehensive Cancer Network Guidelines, version 3.2019, definition: prior bilateral oophorectomy (based on date of surgery); ≥60 years of age; 60 years of age and amenorrheic for ≥12 months in the absence of chemotherapy, tamoxifen, toremifene, or ovarian suppression. Perimenopausal status was captured if it was stated in the clinician notes. Endocrine sensitivity was defined as ≥12 months without recurrence/progression after completion of endocrine therapy in the adjuvant setting. Visceral disease sites included liver, lung, peritoneum, or pleural nodules, consistent with prior palbociclib studies [9,19]. 

#### 2.3.2. Treatment Patterns

Time to chemotherapy initiation was defined as time from the start of first-line therapy with palbociclib plus an aromatase inhibitor to the day before the start of subsequent chemotherapy for patients with evidence of chemotherapy or death for any reason, whichever came first. If a patient did not have evidence of subsequent chemotherapy and did not die, the patient was censored at the date of last contact or end of the study period, whichever came later.

Time to dose adjustment (TTDA) was defined as the length of time from the start of first-line therapy with palbociclib plus an aromatase inhibitor to the date of first-line therapy dose adjustment. 

Time to treatment discontinuation was defined as the length of time from the start of first-line therapy with palbociclib plus an aromatase inhibitor to the earliest of one the following: date the patient discontinued first-line therapy (end of first-line therapy) or date of death. Patients alive and without first-line therapy discontinuation were censored at the earliest of last known use of first-line therapy or end of the study period [25].

#### 2.3.3. Effectiveness Outcomes

Real-world PFS was defined as the length of time from the start of first-line therapy with palbociclib plus an aromatase inhibitor to the earliest of the following: clinician-assessed progression event or date of death. Patients alive and without a progression were censored at the date of initiation of the second line of therapy for patients with 1 line of therapy, the date of last contact with a healthcare provider, or at the end of the study period. A clinician-assessed progression event was defined based on documentation including medical oncology consult/notes, radiation oncology notes, progress notes, discharge summaries, nursing notes, and external reports. 

Overall survival was defined as the length of time from the start of first-line therapy with palbociclib plus an aromatase inhibitor to the date of death. If death did not occur during the study time period, the patient was censored at the end of the study or date of last contact, whichever occurred first [25]. 

### 2.4. Statistical Analyses

Descriptive analyses were used to describe demographic and clinical characteristics at the time of A/MBC diagnosis, with categorical variables reported as frequency and percentage and continuous variables reported as median, minimum, maximum, and interquartile range (IQR). Kaplan-Meier survival analyses were used to descriptively analyze the time-to-event outcomes of rwPFS, OS, TTC, and TTD. 

Subgroups examined for rwPFS included age, stage at diagnosis, Eastern Cooperative Oncology Group (ECOG) performance status, Charlson Comorbidity Index (CCI) score, disease-free interval, race, and number and type of metastatic sites. All data analyses were performed in R version 3.6.1.

## 3. Results

### 3.1. Patients

Between 3 February 2015, and 31 July 2019, a total of 242 patients with HR+/HER2− A/MBC initiated palbociclib plus an aromatase inhibitor as first-line therapy (Figure 1). The median time from start of first-line therapy to the end of follow-up was 22.4 months (IQR, 13.1–33.7). Among all patients, 238 were women and 4 were men; median age at A/MBC diagnosis was 66.0 years; 81.0% (*n* = 196) were White, and 12.0% (*n* = 29) were Black or African American (Table 1). Most patients (85.5%) were postmenopausal, and 10.7% were pre/perimenopausal; menopausal status was unknown or not applicable in 3.7% of patients. 

At initial breast cancer diagnosis, 56.6% of patients (*n* = 137) had advanced/metastatic disease (advanced, *n* = 3; de novo metastatic, *n* = 134), and 43.4% (*n* = 105) had early stage disease (Table 2). Most patients had 1 metastatic site (60.7%; *n* = 147); 50.8% (*n* = 123) had bone-only disease; and 32.2% (*n* = 78) had visceral disease. In total, 47.5% of patients (*n* = 115) had an ECOG performance status score of 0 or 1, and 40.5% (*n* = 98) had an unknown score. Nearly half of patients had hypertension (48.8%; *n* = 118), 21.9% (*n* = 53) had diabetes, and 65.3% (*n* = 158) had a CCI score of 0. Among patients with early stage disease at diagnosis, 60.0% (n = 63/105) received chemotherapy in the adjuvant setting. Of the 80 patients with early stage diagnosis who received endocrine therapies in the adjuvant setting, 53 had available data on the start and end date of systemic therapy; of these patients, 84.9% (*n* = 45) had endocrine sensitivity in the adjuvant setting. 

### 3.2. Treatment Patterns

**Figure 2:**
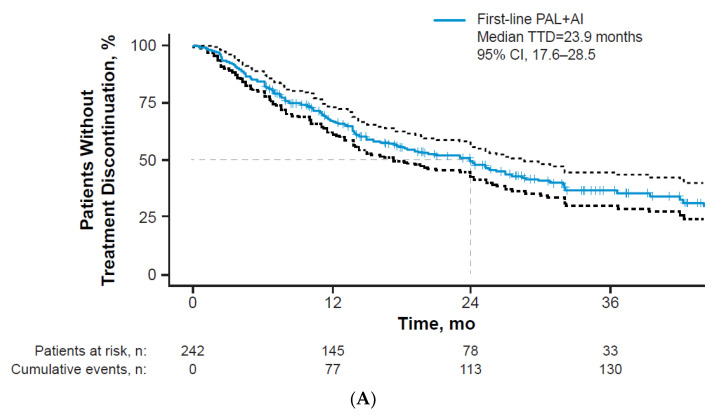

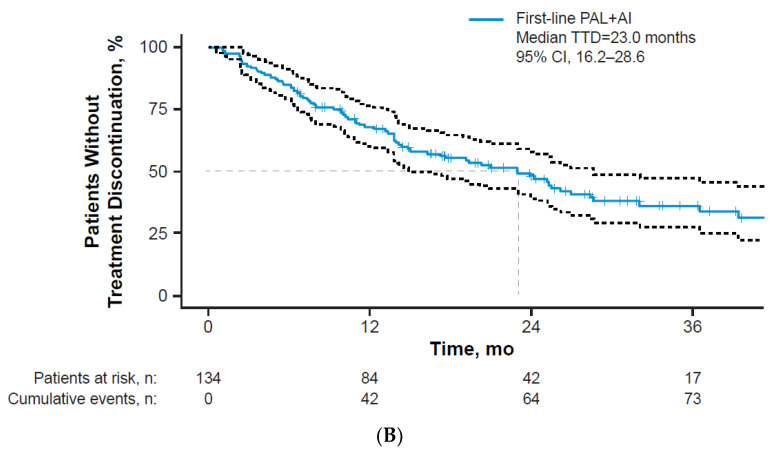
Kaplan-Meier curve of time to treatment discontinuation (**A**) among all patients and (**B**) among patients with stage IV disease at breast cancer diagnosis. AI = aromatase inhibitor; PAL = palbociclib; TTD = time to treatment discontinuation.

The time from A/MBC diagnosis to the start of first-line therapy was ≤30 days for 65.3% of patients and 30 days for 34.7% of patients. A total of 89.7% of patients (*n* = 217) initiated palbociclib at the 125 mg dose. Among all patients, 31.4% had a known dose adjustment in first-line therapy during the study period; most of the adjustments occurred within the first 12 months of treatment (TTDA: median, 55.0 [IQR, 27.8–153.8] days; mean, 167.4 days). Of all patients, 26.4% discontinued due to disease progression and 14.9% due to intolerance or toxicity. The median (95% CI) TTD was 23.9 (17.6–28.5) months among all patients (Figure 2A). In a sensitivity analysis of only patients with stage IV disease at breast cancer diagnosis (*n* = 134), the TTD was 23.0 (16.2–28.6) months (Figure 2B). Dosing information (including reasons for discontinuation and dose adjustments) is presented in Table 3.

At the end of the study, 106 patients (43.8%) were still receiving first-line therapy. Among patients who received a subsequent therapy, 52 patients received a total of two lines of therapy; 31 patients received three lines; 5 patients received four lines; and 8 patients received five or more lines. The most common second-line regimens were a CDK4/6 inhibitor plus endocrine therapy (39.6%) and chemotherapy (20.8%). Among patients who received a second-line regimen (*n* = 96), 16 (16.7%) received a CDK4/6 inhibitor plus fulvestrant; 11 (11.5%) received palbociclib plus fulvestrant. Among all patients who received a CDK4/6 inhibitor as part of any second-line therapy regimen (*n* = 42), 31 (73.8%) received palbociclib; 9 patients (21.4%), abemaciclib; and 2 patients (4.8%), ribociclib. Median (95% CI) TTC was 44.1 (36.6–not estimable (NE)) months.

### 3.3. Clinical Effectiveness

**Figure 3:**
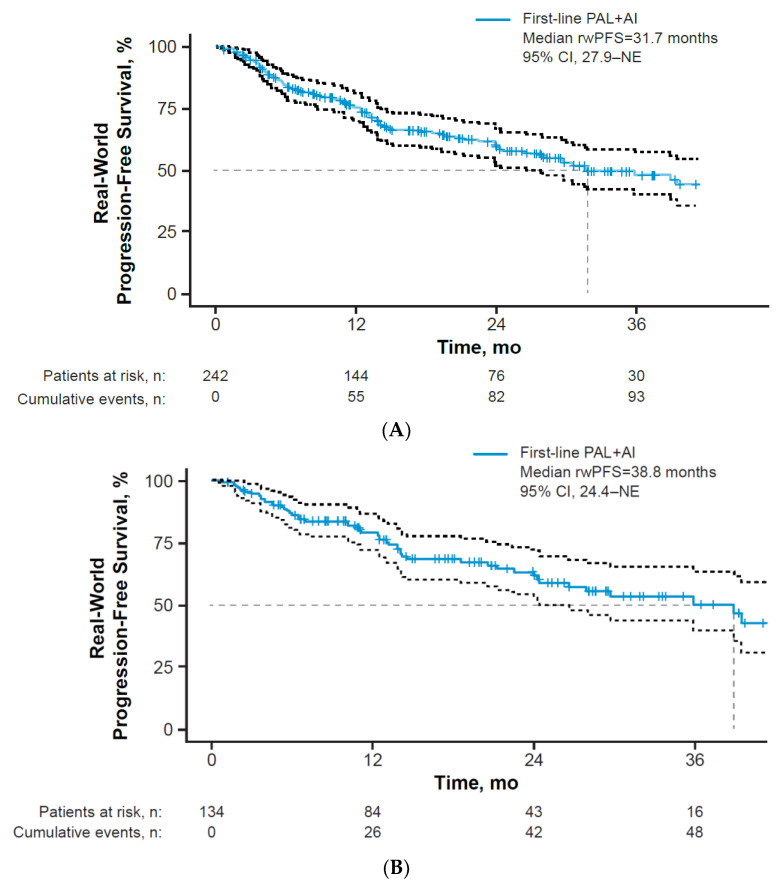
Kaplan-Meier curve of real-world progression-free survival (**A**) among all patients and (**B**) among patients with stage IV disease at breast cancer diagnosis. AI = aromatase inhibitor; PAL = palbociclib; rwPFS = real-world progression-free survival.

Among all patients treated with palbociclib plus an aromatase inhibitor as first-line therapy, median (95% CI) rwPFS was 31.7 (27.9–NE) months (Figure 3A). In a sensitivity analysis of only patients with stage IV disease at breast cancer diagnosis (*n* = 134), median rwPFS was 38.8 (24.4–NE) months (Figure 3B). Median rwPFS by subgroup is presented in Table 4. Patients with bone-only metastatic disease had a longer median (95% CI) rwPFS (44.9 (39.4–NE) months) than did patients with visceral disease (27.9 (13.8–NE) months). In addition, patients with advanced or de novo metastatic breast cancer at initial breast cancer diagnosis achieved a greater rwPFS benefit (38.8 (26.5–NE) months) than patients with early stage breast cancer at initial diagnosis (30.5 (17.4–NE) months; stages 0, I, II, IIIa).

In total, 25.6% of patients died during the study period. At 2 years, the estimated OS rate was 78.0%. However, these data are limited due to a small sample population and by insufficient follow-up time to reliably estimate median OS. 

Of patients who initiated palbociclib at 125 mg (*n* = 217), the median (95% CI) rwPFS was 31.7 (26.5–NE) months. A similar median PFS was observed among patients who initiated palbociclib at 125 mg (*n* = 25; 38.8 (10.1–NE) months).

## 4. Discussion

This retrospective real-world study utilized the Syapse LHN to identify patients with HR+/HER2− A/MBC who were treated with palbociclib plus an aromatase inhibitor as first-line therapy. Findings from this study indicate treatment patterns and effectiveness outcomes with palbociclib plus aromatase inhibitor combination therapy are similar to what has been observed in other EHR real-world studies and complement data from randomized controlled trials [8,9,11,19,26,27,28,29,30]. After a median follow-up duration of 22.4 months, median rwPFS was 31.7 months and the estimated OS rate at 2 years was 78.0%. Among patients with stage IV disease at diagnosis, median rwPFS was 38.8 months. Although subgroup rwPFS data are limited by the small subgroup sample sizes, some trends observed here are consistent with other reported data. For example, patients with better prognostic factors such as bone-only metastases and de novo metastatic disease seem to derive a greater benefit from palbociclib in the first-line setting [11]. Although OS data were limited by insufficient follow-up time and further evaluation is warranted, a recent study of mortality data sources utilized by the Syapse LHN demonstrated a combined mortality composite score with 94.9% sensitivity in the real-world setting, establishing the Syapse LHN as a valid source for death data [24]. Median TTD (23.9 months) was shorter than rwPFS, likely because patients could discontinue treatment for reasons other than progression (i.e., intolerance/toxicity). Discontinuation due to toxicity was reported in about 15% of patients, which is similar to real-world findings in the Flatiron database (~11%) among patients with HR+/HER2− A/MBC who received palbociclib plus an aromatase inhibitor as first-line therapy [31]. In summary, these data from the Syapse LHN add to the body of evidence that demonstrates the real-world benefit of palbociclib combination therapy with an aromatase inhibitor in routine US clinical practice.

Previous real-world studies showed a median rwPFS between 15.1 and 37.9 months with palbociclib combination therapy as first-line therapy for HR+/HER2− A/MBC [19,26,27,28,29,30]. The median rwPFS outcome observed in the current real-world study is longer than some PFS estimates previously reported; this may be due in part to the better overall prognostic characteristics of the patients in this cohort, with a larger proportion of patients with de novo disease and bone-only disease, which tends to be associated with longer PFS [32]. In addition, nuances in rwPFS between studies may also be due to differences in median duration of follow-up; some real-world studies reported between 9.9 and 10.8 months of follow-up [20,26,28]. Moreover, “date of last contact” in the current study was defined as contact by a healthcare provider, and although that provider was likely an oncologist, it was not a requirement. Real-world PFS among patients who were censored as a result of “date of last contact” (i.e., patients were still known to be alive but were not being assessed for progression) was not estimated and is a potential limitation of this study. However, it should not have affected the overall rwPFS since only 5% of all patients were censored due to “date of last contact” and because it would be unlikely to miss an oncologist-identified progression event in patient charts. Furthermore, previous real-world studies include varied patient populations, defined by differences in patient demographic and clinical characteristics (e.g., age, disease burden, and metastatic sites), and used EHRs with different capabilities or healthcare clinic/system data sources across various regions in the United States where patient care practices may differ [19,20,26,27,28,29].

Although the current study was conducted in a patient population distinct from that of the PALOMA-2 trial (i.e., patients in the current study were older, and more patients had only 1 metastatic site, bone-only disease, and de novo metastatic disease), the median rwPFS with first-line palbociclib plus an aromatase inhibitor observed herein (31.7 months) was complementary to the median PFS reported in PALOMA-2 (24.8 months) [9,11]. As mentioned previously, the higher median PFS reported in the present study may be attributed to a higher percentage of patients with bone-only disease and de novo metastatic disease at diagnosis [32,33]. While the reasons for the presence of a larger proportion of de novo A/MBC patients in our study population is unclear, we suspect that patients with de novo metastatic disease may be more likely to be treated with palbociclib plus an aromatase inhibitor, while patients who progressed to A/MBC may be more likely to be treated with palbociclib plus fulvestrant. Future studies and analyses would be required to evaluate this hypothesis. The most common reasons for discontinuation in the current study were also in line with the findings in the palbociclib arm of PALOMA-2 (disease progression, 26.4% vs. 38.7%, respectively; toxicity, 14.9% vs. 9.7%) [9]. Treatment sequencing after palbociclib combination therapy is still heterogeneous, with many patients in this analysis receiving CDK4/6 inhibitors as second-line treatment (~40%); not surprisingly, the switch of endocrine partner to fulvestrant was a common change. However, therapy selection following a CDK4/6 inhibitor is not currently defined in treatment guidelines. In summary, real-world studies and randomized clinical trials both provide valuable information on treatment patterns and effectiveness outcomes among different populations of patients and within different study settings. Future studies examining patient characteristics associated with receipt of palbociclib plus aromatase inhibitor among the first-line treated patients could further enhance our understanding of clinical practice in this cohort of patients in the Syapse Learning Health Network and predominantly Midwestern community health systems.

Limitations of this study include its single-arm nature and the small sample size of the overall population and, subsequently, sample sizes of the subgroups; these small sample sizes hinder the definitive interpretation of the results. At the time of this study, health systems with EHR data for abstraction were predominantly located in the Midwest United States. Thus, approximately 95% of patients originated from the Midwest, and patient care practices may differ outside of this region. Real-world studies are also limited by missing data (e.g., elements that were not able to be abstracted or were not captured in the patient records, such as ECOG performance status in this study, for which specific medical record documentation has been previously observed in approximately 50% of patients [34] and attributed to lack of consistent medical record documents) or erroneous data entry. 

## 5. Conclusions

This study utilized a US EHR data source that has not been previously used to assess palbociclib treatment patterns and outcomes in A/MBC. It included a population of patients not included in other EHRs with varying disease burden and baseline characteristics, which may account for the differences/nuances observed in clinical effectiveness outcomes. This is also the first study of palbociclib use in a patient cohort from large community health systems that provide integrated care (oncology and non-oncology care), in contrast to prior studies assessing patients treated in an academic or independent community practice setting. Overall, these data complement other real-world studies of palbociclib that utilized other EHR databases, as well as support findings observed in randomized controlled trials of palbociclib. Dosing patterns and discontinuation rates were consistent with previous data, and palbociclib plus aromatase inhibitor combination therapy continues to be well-tolerated by patients with HR+/HER2− A/MBC. In summary, these data support the continued use of palbociclib plus an aromatase inhibitor as first-line therapy for patients with HR+/HER2− A/MBC. 

## Figures and Tables

**Table 1:** Patient demographics and clinical characteristics (*n* = 242).

Characteristic	*n* (%)
**Sex**	
Female	238 (98.3)
Male	4 (1.7)
**Race**	
White	196 (81.0)
Black or African American	29 (12.0)
Asian	10 (4.1)
Other or not provided	7 (2.9)
**Ethnicity**	
Hispanic-Latino	15 (6.2)
Non-Hispanic/Non-Latino	225 (93.0)
Unknown	2 (0.8)
**Region of residence**	
Midwest	230 (95.0)
South	10 (4.1)
Other	2 (0.8)
**Age at A/MBC diagnosis, y**	
Median (min, max)	66.0 (31.0–93.0)
50	25 (10.3)
50–64	86 (35.5)
65–74	80 (33.1)
75	51 (21.1)
**Menopausal status at A/MBC diagnosis**	
Postmenopausal	207 (85.5)
Pre/perimenopausal	26 (10.7)
Unknown/not applicable *	9 (3.7)
**Insurance carrier**	
Medicare/Medicaid	149 (61.6)
Commercial	69 (28.5)
None/Not stated	24 (9.9)

A/MBC = advanced/metastatic breast cancer. * “Not applicable” menopausal status refers to male patients.

**Table 2:** Disease characteristics and comorbidities (*n* = 242).

Characteristic	*n* (%)
**A/MBC at diagnosis**	137 (56.6)
**Early stage at diagnosis**	105 (43.4)
**Setting where first-line palbociclib plus AI was received**	
Advanced Setting	6 (2.5)
Metastatic Setting	236 (97.5)
**ECOG PS**	
0	57 (23.6)
1	58 (24.0)
≥2	29 (12.0)
Unknown	98 (40.5)
**CCI Score**	
0	158 (65.3)
1	47 (19.4)
2	22 (9.1)
3	9 (3.7)
≥4	6 (2.5)
**Specific comorbidities**	
Hypertension	118 (48.8)
Diabetes	53 (21.9)
Renal disease	22 (9.1)
Chronic pulmonary disease	14 (5.8)
None	89 (36.8)
DFI * in patients with early stage disease at diagnosis, mo (*n* = 105)	
12	14 (13.3)
13–24	12 (11.4)
25–36	10 (9.5)
36	69 (65.7)
**Number of metastatic sites ^†^**	
0	1 (0.4) ^‡^
1	147 (60.7)
2	45 (18.6)
≥3	49 (20.2)
**Sites of distant metastases ^§^**	
Bone (bone only or in addition to other sites)	201 (83.1)
Bone only	123 (50.8)
Visceral ^||^	78 (32.2)
Lung	52 (21.5)
Brain	6 (2.5)
Distant lymph nodes	45 (18.6)
Liver	24 (9.9)
Malignant pleural effusion	25 (10.3)
**BRCA 1/2 tested**	
Yes	46 (19.0)
No	196 (81.0)

A/MBC = advanced/metastatic breast cancer; AI = aromatase inhibitor; CCI = Charlson Comorbidity Index; DFI = disease-free interval; ECOG PS = Eastern Cooperative Oncology Group performance status. * Date of end of adjuvant treatment to disease recurrence; ^†^ One organ system can have multiple lesions/sites but it was classified as only 1 metastatic site. ^‡^ Index date was the start of first-line palbociclib plus an aromatase inhibitor after advanced or metastatic disease diagnosis; 6 patients received first-line palbociclib plus an aromatase inhibitor after advanced disease diagnosis and 1 of these patients was not metastatic at the end of study. ^§^ Sites of distant metastases in 5% of patients and among patients with brain metastases are shown. Sites of distant metastases in ≤5% of patients include central nervous system, contralateral breast, peritoneum, pleural nodules, skin, ovary, adrenal, bone marrow, colon, omentum, soft tissue, stomach, and other. ^||^ Visceral sites include liver, lung, peritoneum, and pleural nodules.

**Table 3:** Palbociclib dosing information (*n* = 242).

Dosing Information	*n* (%)
**Starting dose, mg**	
125	217 (89.7)
100	18 (7.4)
75	3 (1.7)
Unknown	4 (1.2)
**End dose, mg**	
125	147 (60.7)
100	67 (27.7)
75	24 (9.9)
Unknown	4 (1.7)
**Dose adjustment**	
None	162 (66.9)
Decrease	75 (31.0)
Increase	1 (0.4)
Unknown	4 (1.7)
**Treatment ongoing**	
Yes	106 (43.8)
No	136 (56.2)
**Discontinuation reason**	
Progression	64 (26.4)
Intolerance/toxicity	36 (14.9)
Other *	36 (14.9)

* Includes patient choice, changes in insurance or health systems, physician choice, hospice referrals, death, end of planned therapy, treatment for other disease, actionable mutation found, and other/unknown.

**Table 4:** Real-world progression-free survival by subgroup.

Subgroup	*n*	Median rwPFS (95% CI)
**Metastatic disease sites**		
Bone only	123	44.9 (39.4–NE)
Visceral	78	27.9 (13.8–NE)
**Stage at breast cancer diagnosis**		
Advanced or metastatic (stages IIIb, IIIc, IV)	137	38.8 (26.5–NE)
Metastatic (stage IV)	134	38.8 (24.4-NE)
Early stage (stages 0, I, II, IIIa)	89	30.5 (17.4–NE)
Unknown	16	29.8 (23.9–NE)
**Age at A/MBC diagnosis, y**		
50	25	NR (13.3–NE)
50–64	86	26.5 (17.4–NE)
65–74	80	41.9 (29.8–NE)
≥75	51	35.8 (21.2–NE)
**ECOG PS**		
0	57	29.8 (27.9–NE)
1	58	31.7 (19.4–NE)
2+	29	13.8 (5.7–NE)
Unknown	98	38.8 (30.5–NE)
**CCI score**		
0	158	44.9 (28.0–NE)
1	47	26.5 (13.3–NE)
2+	37	29.8 (21.2–NE)
**DFI among patients with early stage disease at diagnosis, mo**		
≤12	14	13.3 (3.7–NE)
12	91	31.6 (23.9–NE)
**Race**		
White	196	35.8 (24.4–NE)
Black or African American	29	18.5 (13.8–NE)
Number of metastatic sites *		
1	147	44.9 (29.6–NE)
2	45	31.6 (14.0–NE)
≥3	49	14.7 (12.3–NE)

A/MBC = advanced/metastatic breast cancer; CCI = Charlson Comorbidity Index; DFI = disease-free interval; ECOG PS = Eastern Cooperative Oncology Group performance status; NE = not estimable; NR = not reached; rwPFS = real-world progression-free survival. * Excludes 1 person who was not metastatic.

## Data Availability

Upon request, and subject to review, Pfizer will provide the data that support the findings of this study. Subject to certain criteria, conditions and exceptions, Pfizer may also provide access to the related individual de-identified participant data. See https://www.pfizer.com/science/clinical-trials/trial-data-and-results for more information (1 February 2022).
